# Aronia in the Type 2 Diabetes Treatment Regimen

**DOI:** 10.3390/nu15194188

**Published:** 2023-09-28

**Authors:** Christine B. Christiansen, Per B. Jeppesen, Kjeld Hermansen, Søren Gregersen

**Affiliations:** 1 Department of Clinical Medicine, Aarhus University, Palle Juul-Jensens Boulevard 11, 8200 Aarhus N, Denmark; per.bendix.jeppesen@clin.au.dk (P.B.J.); kjeld.hermansen@clin.au.dk (K.H.); soeren.gregersen@aarhus.rm.dk (S.G.); 2 Department of Endocrinology and Internal Medicine, Aarhus University Hospital, Palle Juul-Jensens Boulevard 99, 8200 Aarhus N, Denmark; 3 Steno Diabetes Center Aarhus, Palle Juul-Jensens Boulevard 11, 8200 Aarhus N, Denmark

**Keywords:** Aronia melanocarpa, type 2 diabetes, dietary supplement, antioxidants, polyphenols, anthocyanins, randomized controlled trial

## Abstract

Aronia melanocarpa berries are rich in antioxidants and possess a high antioxidant capacity. Aronia berries have shown potential in type 2 diabetes mellitus (T2DM) treatment, and previous studies indicate improvements in glycemia after supplementation. Unfortunately, the effectiveness of aronia berries is limited by the low bioavailability of aronia, which fermentation could potentially overcome. The objective of this study was to compare the effects of fermented or non-fermented aronia pulp with placebo in subjects with T2DM. This study was a triple-blinded, triple-crossover study with eight-week intervention periods with fermented aronia extract (FAE), non-fermented aronia extract (AE), and placebo. Extracts were incorporated in snack bars with 37% aronia (FAE or AE) or wheat bran (placebo) and 63% raisins and coconut oil. Pre- and post-treatment period, we did fasting blood samples, including hemoglobin A1c, fructosamine, insulin, glucose, glucagon-like peptide-1, glucose-dependent insulinotropic peptide (GIP) and glucagon, oral glucose tolerance tests, and anthropometric measurements. Of 36 randomized participants, 23 completed the trial. Aside from a higher increase in GIP after FAE supplementation compared to after placebo supplementation, aronia extracts had no effect. The increase in GIP levels after FAE supplementation may hold potential benefits, but the overall clinical impact remains unclear.

## 1. Introduction

Aronia melanocarpa is a garden shrub that is convenient to cultivate and requires low maintenance [1]. It sets berries that have a high content of anthocyanins. Interestingly, they apparently have potential as a treatment for type 2 diabetes mellitus (T2DM) [2]. Two clinical studies have been conducted with aronia in individuals with T2DM [3,4]. Unfortunately, the studies were not blinded, nor randomized [3,4]. Milutinovic et al. [4] tested the effect of three daily doses of 50 mL aronia juice, corresponding to 258 mg anthocyanins, for three months and compared the results to no supplementation for three months in 35 T2DM patients. They observed a significant reduction in LDL cholesterol after aronia supplementation, but only non-significant reductions in fasting blood glucose, hemoglobin A1c (HbA1c), total cholesterol, and triglycerides. In contrast, Simeonov et al. found that the administration of 200 mL aronia juice twice daily for three months resulted in a significant reduction in fasting blood glucose, HbA1c, and cholesterol, along with a non-significant improvement in blood pressure [3]. Similarly, an acute study in healthy subjects found a significant reduction in postprandial blood glucose when 100 mL of aronia juice was administered before a meal [5].

Aronia berries have attained great interest in relation to their high antioxidant capacity [2,6]. This high antioxidant capacity is attributed to aronia’s high content of polyphenols such as anthocyanins, procyanidins, and phenolic acids [6]. As oxidative stress is increased in T2DM, it has been suggested that antioxidants may have protective effects and can reduce hyperglycemia [7]. In relation, several in vitro studies have demonstrated aronia’s strong antioxidant potential [8,9,10,11]. To assess aronia’s antioxidant potential in humans, a meta-analysis has examined its effect on exercise-induced oxidative stress, but the results are conflicting [12]. Although aronia berries have a high antioxidant capacity in vitro [8], we do not know whether health-promoting mechanisms are of clinical relevance in humans [13]. However, there is further evidence that aronia berries may have beneficial effects on T2DM through other mechanisms, e.g., via enzyme-regulating abilities. In regard to enzyme regulation, aronia berries are capable of inhibiting α-glucosidase and dipeptidyl peptidase 4 in vitro [5] and in vivo in diabetic KKAy mice [14]. Antidiabetic agents with such properties are marketed. α-glucosidase inhibition delays the breakdown of disaccharides in the small intestine and thus the release of glucose into the bloodstream [15]. Inhibition of dipeptidyl peptidase 4 prevents degradation of glucagon-like-peptide-1 (GLP-1) and glucose-dependent insulinotropic polypeptide (GIP) [16]. Both GLP-1 and GIP in turn increase insulin secretion, while only GLP-1 decreases glucagon secretion, which leads to lower blood glucose [16]. In addition, in vitro models, which use cell lines that possess characteristics similar to liver and skeletal muscle cells, have demonstrated that aronia berries can improve insulin sensitivity by increasing the expression of glucose transporter 4 [17,18]. These findings are in line with findings in two in vivo animal studies of T2DM that found a beneficial effect of aronia on insulin sensitivity and on insulin and glucose concentrations [19,20].

It has been reported that the polyphenols from aronia berries are poorly absorbed, which may limit their health-promoting effects [21,22]. However, this obstacle may be solved by fermentation. Studies have shown that fermentation enhances the bioavailability of antioxidants [23,24] and increases the α-glucosidase inhibition [24]. Furthermore, an in vivo study where mice received a high-fat diet along with either aronia berries or fermented aronia berries showed superior effects of fermented aronia, particularly regarding insulin and glucose sensitivity [25].

Therefore, we wanted to further clarify the role of aronia in the treatment of T2DM. At present, no clinical studies have examined the effect of aronia in a blinded setting or investigated the effect of aronia on glucose metabolism and on circulating levels of glucagon, incretin, and insulin. Thus, the objective of the present study was to compare the effects of eight weeks of administration of fermented or non-fermented aronia pulp with a placebo in a blinded setting in subjects with T2DM. We hypothesized that both aronia extracts would improve glucose metabolism, however, the fermented more than the unfermented product, in comparison to placebo, with AUC and iAUC during OGTT being the primary endpoint. Secondary outcomes include computations of glucose metabolism and measurements of circulating levels of glucagon, incretin, and insulin.

## 2. Materials and Methods

### 2.1. Study Design

The trial was a triple-blinded, triple-crossover study with intervention periods of eight weeks duration. Between each intervention period, there was a wash-out period lasting at least three weeks. Each participant received fermented aronia extract (FAE), aronia extract (AE), or placebo in a random order. The dietary supplements were consumed twice daily, once in the morning, before breakfast and once in the evening, before dinner. The participants were randomly assigned to the treatment order using block randomization based on random sequence generation in Excel. A person not related to the study generated the codes and put them in sealed envelopes. Participants were recruited through advertisements in local newspapers, at a dedicated website for recruitment of participants (“forsoegsperson.dk”), and on social media platforms. The study candidates received study information orally and in writing and were given one week for consideration. If the candidate was interested in participating, a written consent was obtained. Participants had the right to withdraw their participation at any time.

All tests and examinations were performed before and after each of the three intervention periods, e.g., a total of six repeats. Tests and examinations included fasting blood samples, oral glucose tolerance tests, and measurements of weight and waist circumference. The study was conducted at the Department of Endocrinology and Internal Medicine, Aarhus University Hospital, Denmark from December 2020 to April 2022. The trial was performed in accordance with the Declaration of Helsinki and approved by The Danish Ethics Committee (Journal no. 1-10-72-102-19). The trial was registered at ClinicalTrials.gov under NCT04647175.

### 2.2. Participants

Inclusion criteria were diagnosis of T2DM, age of 30 to 80 years, fasting blood glucose ≤ 12 mmol/L, and HbA1c > 6.1% and <10% (>43 and <86 mmol/mol) if participants were in medical diabetes treatment OR HbA1c > 6.5% and <10% (>48 and <86 mmol/mol) if participants were treated with lifestyle only. Any treatment modality was accepted, including GLP-1 agonists and insulin therapy. Exclusion criteria were changes in diabetes medication within the last 3 months, serious comorbidities, including cardiovascular, neurological, psychological, and/or renal diseases, alcohol or substance abuse, and pregnant or planned pregnancy. To make an overall health assessment of the participants before enrolment, various other blood tests were performed. The participants should abstain from aronia consumption three weeks prior to trial initiation. During the trial, they were not allowed to consume other aronia products than the test supplements.

### 2.3. Test Products

The test products are made on leftover aronia pulp from juice production and kindly provided by the company Elkærholm (Egtved, Denmark). To reduce variations in the test products’ antioxidant potential due to differences in cultivation conditions, all test products were based on berries harvested at the same time and location. In order to make the aronia pulp extract edible, snack bars consisting of aronia, raisins, and coconut oil were produced. For FAE and AE, a daily dose was composed of 34 g of either fermented or non-fermented aronia, 55 g of raisins, and 3 g of coconut oil. In the placebo bars, aronia pulp was substituted with 17 g of wheat bran and 5 g of liquid (water and colorants). The anthocyanin concentration in both fermented and non-fermented aronia was assessed by Elkærholm using HPLC, which measured the combined concentrations of cyanidin-3-galactoside, cyanidin-3-arabinoside, and cyanidin-3-glucoside. A daily dose of 34 g of fermented aronia contained 893 mg of anthocyanins, while the same quantity of non-fermented aronia provided 533 mg of anthocyanins.

HPLC measurements were made on berries harvested in 2018, whereas berries from 2019 were used for the test products. The pH differential method was used to determine the relative cyanidin-3-glucoside concentration in both fermented and non-fermented aronia made from berries harvested in both 2018 and 2019. The cyanidin-3-glucoside equivalent anthocyanin concentration was higher in the aronia pulp extract from berries from 2019 than in 2018, by 3.3 and 2.12 times for non-fermented and fermented aronia, respectively. It should be emphasized that natural decay during storage undoubtedly contributed to this difference.

Energy contents of the test products were measured by Eurofins Steins Laboratorium (Vejen, Denmark). The nutritional composition of the three test products was comparable as outlined in Table 1. Test products were stored at −21 °C. Before each intervention period, the participants were handed over the test products for eight weeks. The participants were instructed to store the test products for the first four weeks in their refrigerator at approximately 5 °C and to store the remaining test products in the freezer at approximately −21 °C until usage. The test products were handed over in sealed brown bags that had been coded by a person not related to the study. The code was not revealed until after the data analysis.

### 2.4. Compliance

Each daily dose of test product (2 bars) was individually packed in plastic bags. In order to assess compliance, the participants handed in empty plastic bags after each 8-week intervention period, which were then counted by blinded personnel.

### 2.5. Oral Glucose Tolerance Test

The last supplement was consumed the evening before the OGTT allowing for an eight-hour fast prior to the OGTT. At the OGTT, the participants consumed 75 g of glucose dissolved in 150 mL of water within 5 min. Blood was sampled from a peripheral venous catheter placed at the antecubital fossa at time points −10, 0, 30, 60, 90, 120, and 240 min.

### 2.6. Anthropometric Measurements

Body weight measurements were determined using a calibrated SECA scale. For each participant, the same scale was used at all visits. Waist circumference was determined using a measuring tape. The same investigator undertook all measurements.

### 2.7. Blood Analyses

Blood was collected from participants after an eight-hour fast. HbA1c was measured in whole blood immediately after collection. Baseline values were also measured immediately. Remaining samples were centrifuged at 4 °C and 3989 RPM for 10 min after which plasma was stored at −80 °C until analysis.

#### 2.7.1. Baseline Measurements, HbA1c and Lipids

Baseline measurements and HbA1c concentrations were measured by Department of Clinical Biochemistry at Aarhus University Hospital, Denmark (DS/EN ISO 15189:2013 approved).

#### 2.7.2. Glucose (Complete Cases)

For participants that completed the trial, glucose was quantified on Cobas c111-system using a commercially available enzymatic colorimetric kit (#11491253 216, Roche Diagnostics, Basel, Sitzerland) that employs a glucose oxidase–peroxidase method. Intra-assay and inter-assay coefficients of variability (CV) were between 1.25–2.18% and 2.29–2.40%, respectively. For incomplete cases, glucose concentrations were measured as described below.

#### 2.7.3. Fructosamine and Glucose (Incomplete Cases)

The Indiko analyzer was used with commercially available kits to determine blood concentrations of fructosamine (#FR4030, Randox Laboratories Ltd., Malling, Denmark) for all participants and glucose (#981779, ThermoFisher Scientific, Roskilde, Denmark) for the non-completers.

#### 2.7.4. Insulin, Glucagon, GIP, and GLP-1

Commercially available ELISA kits were used to determine the concentrations of insulin (#10-1113-01, MERCODIA), glucagon (#10-1271-01, MERCODIA), GIP (#10-1258-01, MERCODIA) and GLP-1 (#10-1278-01, MERCODIA). The concentration of glucagon was below the lower limit of detection in 11 samples. These values were dispersed across all groups. Seven values were below the lower limit of detection for GIP; again, values were dispersed across all groups.

### 2.8. Power Calculation

The power calculation was performed for our primary endpoint, which was a 240 min incremental area under the curve (iAUC) for glucose during an OGTT. In a previous study investigating the effect of stevioside in T2DM, we identified a minimally relevant difference for a 240 min iAUC for a test meal of 18% (iAUC 638 ± 55 versus 522 ± 64 mmol/L × 240 min (control versus stevioside); *p* < 0.02) [26]. The number of participants needed to obtain a statistical power of 80% at a level of *p* < 0.05 (α = 0.05; 1−β = 0.8) was calculated as 18. With an anticipated dropout rate of 20%, we aimed to enroll 23 participants. However, based on the actual dropout rate early in the study, we increased the number to 36.

### 2.9. Statistics and Calculations

For baseline data, normality was assessed visually by histograms and by Shapiro–Wilk test using RStudio (version 2023.06.1). Normally distributed data are presented as mean ± standard deviation (SD) and non-normally distributed data are presented as median (interquartile range (IQR)). Total area under the curve (AUC) and iAUC for glucose and insulin during the OGTT were calculated with GraphPad Prism (Version 9). In order to assess insulin sensitivity, homeostasis model assessment for insulin resistance (HOMA-IR) [27] and Matsuda indices were calculated [28]. Matsuda indices were calculated for the 120 min area under the glucose and insulin response curves (AUC) in order to enable meaningful comparisons with findings from the existing literature. For comparisons, data were analyzed using a linear mixed effects model with order, period, treatment, and time, along with the interaction between treatment and time, as fixed effects. Record id and record id within the period were included as random effects. The R package lme4 was used for the model. Record id is unique for each participant, order has 6 levels and refers to the order of the treatment, period refers to if results are from the first, second, or third treatment period and time has two levels: pre and post. In order to account for the usage of two different machines to measure blood glucose, the systemic effect of a machine with two different levels was included in the model when assessing blood glucose levels.

Normality was evaluated using quantile–quantile plots of residuals, fitted values versus residuals plots, and histograms of residuals. The RStudio package emmeans was used to obtain estimated marginal means, which will be referred to as mean throughout the article, and to compare means. Normally distributed data are presented as the difference between pre- and post-values with standard error. Not normally distributed data were log-transformed, and they are presented as ratio and confidence intervals, e.g., 1.02 indicates a 2% increase from pre to post, whereas 0.98 indicates a 2% decrease.

In cases where blood analyses revealed concentrations below the lower level of detection for the various assays, the values were computed as half of the lower level of detection.

## 3. Results

### 3.1. Baseline Characteristics

**Figure 1:**
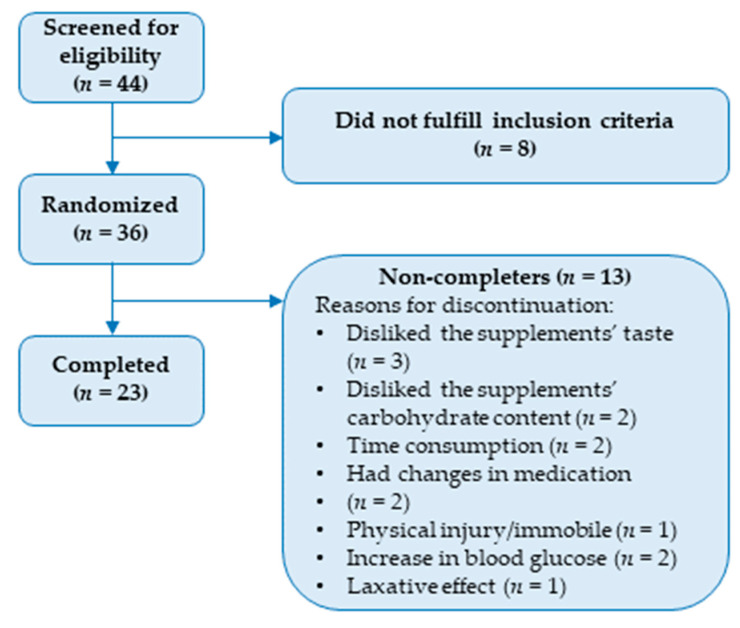
CONSORT flow diagram outlining inclusions, exclusions, and reasons for discontinuation.

Forty-four persons were screened for the trial, of which thirty-six were randomized. The remaining eight did not fulfill the inclusion criteria. A total of 23 participants completed the trial, of which 15 were men and 8 were women. A CONSORT flow diagram, including reasons for exclusion and reasons for discontinuation of the intervention, is presented in Figure 1. Baseline characteristics are summarized in Table 2. In brief, the participants had a mean age of 67.6 ± 5.5 years, a mean body weight of 82.0 ± 16.2 kg, a median body mass index (BMI) of 26.7 (23.2–29.8) kg/m^2^, a mean blood pressure of 141 ± 23.3 mmHg/81 ± 13.3 mmHg, a mean fasting blood glucose of 7.7 ± 1.6 mmol/L, and a median HbA1c of 50 (47.5–54) mmol/mol. In addition, waist circumference was 101.5 (94.4–108.2) cm for men and 115.5 (95.6–124.4) cm for women. An overview of the medications that the participants received during the study is provided in Table 3.

### 3.2. Compliance

Based on the number of empty plastic bags the participants handed in, EMM for compliance was 99% ± 1%, 97% ± 1%, and 95% ± 1% for intervention with FAE, AE, and placebo, respectively. The 3% difference in compliance between FAE and placebo was statistically significant at *p* = 0.01.

### 3.3. Anthropometric Measurements

In regard to anthropometric measurements, as outlined in Table 4, the various interventions had sparse effects on body weight, BMI, and waist circumference. Overall, we observed minor increases of less than one kg body weight and less than one cm in circumference in all intervention groups. After the FAE intervention, we observed a significant increase in body weight of 0.75 ± 0.30 kg (*p* = 0.04). However, BMI remained unchanged during the entire study.

### 3.4. Blood Analyses

HbA1c and fructosamine measurements were applied to evaluate the effect of the interventions on blood glucose over the preceding period of approximately 3 months and 2 weeks, respectively [29]. However, we observed no effect of the interventions. Likewise, there was no effect on fasting insulin, glucose, or glucagon after 8 weeks of intervention. Based on the OGTT, different mathematical formulas were utilized to examine the impact on glucose and insulin resistance. These included the computation of AUC, iAUC, HOMA-IR, and Matsuda. Again, we observed no effect of the interventions on any of these computations. Furthermore, we looked at the effect of the interventions on fasting incretin levels. After 8 weeks of intervention with FAE, GIP levels were significantly increased from an EMM of 5.53 ± 1.01 to 8.81 ± 1.61 (*p* = 0.007). Furthermore, GIP levels were significantly more increased in the FAE intervention group (ratio of 1.59 (1.11–2.29)) compared to in the placebo group where GIP had decreased during the intervention (ratio of 0.94 (0.65–1.34)) (*p* = 0.03). GLP-1 was unaffected by the interventions.

## 4. Discussion

In this study, we investigated the effect of an 8-week supplementation regimen with two different formulations of aronia extract, i.e., fermented and non-fermented formulations, and compared with placebo on glucometabolic parameters in subjects with T2DM. We hypothesized that both aronia extracts would improve glucose metabolism in comparison to placebo, with AUC and iAUC during OGTT being the primary endpoint. We found no significant impact on waist circumference, BMI, HbA1c, fructosamine, or fasting values of insulin, glucose, GLP-1, or glucagon. Additionally, we detected no impact on the AUC of glucose or insulin or influence on insulin sensitivity. However, we did observe a minor increase in body weight and an elevation of the fasting level of GIP in the FAE group. Notably, the increase in GIP was significantly higher after intervention with FAE compared to placebo, whereas no intergroup differences were observed in relation to body weight.

Regarding incretins, it is well-established that GLP-1 analogs improve disease outcomes for individuals with T2DM [30]. However, the role of fasting endogenous incretin levels has not been thoroughly investigated. Since GIP promotes insulin secretion from pancreatic beta-cells [16], the observed increase in GIP may be beneficial in the context of T2DM. On the other hand, the effect of the GIP increase might be without clinical importance since individuals with T2DM in general exhibit severe GIP resistance in the pancreatic beta-cells [31]. Lastly, both in vivo studies and in vitro studies have established that aronia possesses dipeptidyl peptidase 4 inhibiting abilities [5,32], which might account for the observed GIP increase. However, this ability remains to be confirmed in vivo in humans.

We chose to incorporate the aronia extracts in snack bars as we expected higher compliance in comparison to, e.g., pills or capsules. Indeed, we observed an excellent compliance of above 95% for those who completed the trial. Nevertheless, it is possible that the nutritional composition of the bars contributed to the observed slight increases in weight, BMI, and waist circumference after supplementation with FAE and AE. The test products were high in sugar, as raisins were used to make the aronia pulp eatable and improve taste and sensory acceptance. Since it has been demonstrated that the consumption of raisins only produces a minor increase in postprandial blood glucose [33], it was considered a safe choice. Specifically, Esfahani et al. reported a glycemic index of 50 g of raisins of 49 [33], which according to the Brand–Miller classification is categorized as a low glycemic index [34]. In addition, the amount of carbohydrates in the three test products was similar. Nevertheless, the sugar may have masked a possible effect of aronia. Furthermore, our results are in opposition to the findings by Milutinović et al., who observed a significant decrease of 0.4 kg/m^2^ after supplementation with aronia [4].

Additionally, we found no significant differences in fasting and long-term blood glucose control, glucose tolerance, or insulin concentrations following any of the interventions. This is in contrast to results from in vitro and in vivo animal models of T2DM that have reported positive effects on the aforementioned parameters [17,18,19,20]. Our findings are in contrast to findings by Simeonov et al. who observed improvements in both fasting blood glucose and HbA1c after 3 months of supplementation with aronia juice to subjects with T2DM [3]. Furthermore, our previously published systematic review revealed a small but significant reduction of 0.44 mmol/L in fasting blood glucose after aronia supplementation to subjects with cardiovascular disease or risk factors [6]. On the other hand, the results from this study are in line with the findings by Milutinović et al. [4]. Although the investigators observed a trend towards improvement in fasting blood glucose and HbA1c in subjects with T2DM after 3 months of supplementation with aronia juice, no significant differences were found between baseline and 3-month follow-ups [4]. However, the amount of administered aronia was rather low, and it was administered as a supplement to the patient’s regular medication [4]. It is also important to consider that the two aforementioned studies were not blinded, and the improvements they observed in fasting glucose and HbA1c may be attributed to the placebo effect.

It is worth mentioning that overall, the participants in our study were rather well-regulated with fasting blood glucose of ~8.4 mol/L and HbA1c of ~54 mmol/mol. Consequently, it could be questioned whether our participants were too well controlled regarding risk markers to demonstrate any improvements after supplementation. In contrast, Simeonov et al. reported a pre-intervention mean fasting glucose of ~13.3 mmol/L and a mean HbA1c of ~79 mmol/mol, which were subsequently reduced to a level similar to our baseline values after supplementation, resulting in a mean fasting blood glucose of ~9.1 mol/L and HbA1c of ~58 mmol/mol.

One notable strength of our study is the high amount of anthocyanins provided. In comparison, the studies included in our aforementioned systematic review generally employed aronia supplements that typically provided between 300 mg and a maximum of 600 mg polyphenols per day [6]. Our aronia extracts provided that amount in anthocyanins alone. Thus, it is reasonable to assume that the total polyphenolic content was significantly higher in our study. On the other hand, one possible disadvantage is that we only administered two supplements per day. Since anthocyanins from aronia reach their maximum concentration within 3 h and have an estimated half-life of less than 2 h in the bloodstream, administering aronia three times a day or even more might have improved the outcomes of this study [35,36]. On the other hand, the anthocyanins are extensively metabolized, and some metabolites have a half-life of up to 96 h [36]. However, an increase in the number of daily doses could have reduced compliance. Furthermore, we only have anthocyanin measurements on aronia extracts from one time point, and these measurements were not repeated at a later time. As a result, we lack information regarding the anthocyanin content in the snack bars and how it may be affected by factors such as storage, processing, or the food matrix. Furthermore, given the large heterogeneity among individuals with T2DM regarding body composition, medication, blood counts, etc. [37], the cross-over design can also be considered a great advantage. A disadvantage is the dropout rate, which was rather high with only 23 completing the trial of 36 recruited participants. The reasons for participants not completing the study primarily included the study’s duration (*n* = 3), the taste of the supplements (*n* = 3), the carbohydrate content (*n* = 2), and changes in medication (*n* = 2). In addition, three participants experienced adverse effects; one reported a laxative effect, while two observed increases in blood glucose levels. Thus, both the supplements’ taste and the study duration can be considered disadvantageous. Shortening the study duration and improving the taste and nutritional composition of the supplements might have reduced the dropout rate.

We hypothesized that FAE would deliver an increased amount of bioavailable polyphenols, potentially resulting in improved efficacy. It should be considered that fermentation might have the opposite effect and make the polyphenols more susceptible to degradation [38,39]. However, it is plausible that supplementation for a longer period might have revealed different results. Furthermore, FAE and AE are based on aronia pulp while previous studies on the effect of aronia in T2DM used aronia juice [3,4], and even though the pomace, in general, delivers a higher amount of antioxidants [40], the bioavailability might differ between the formulation. Additional HPLC analyses to quantify anthocyanin metabolites in the participants’ blood could have provided valuable insights into the bioavailability.

## 5. Conclusions

In conclusion, our study demonstrated that supplementation with either fermented or non-fermented aronia extract did not affect waist circumference, insulin sensitivity, or fasting levels of glucose, insulin, or glucagon in subjects with T2DM. To our knowledge, this study is the first examination of dietary supplementation with aronia extracts on glucose metabolism in subjects with T2DM. Our research contributes to the expanding body of knowledge on aronia supplementation on human health. However, there is a need for further investigations, particularly on the effect of longer intervention periods as well as a need for studies with a larger sample size.

## Figures and Tables

**Table 1:** Nutritional composition of test products containing aronia extract or placebo. Values are for one daily dose (2 bars). For macronutrients, we also provide energy in percentage in square brackets, which are calculated by assigning 4 kcal/g of carbohydrate or protein and 9 kcal/g of fat and dividing that value by the energy content.

Nutritional Content Per Daily Dose	FAE	AE	Placebo
Energy (kcal)	234.6	240.1	227.3
Total fats (g) (%)	3.7 (14.2)	4.0 (15.0)	3.5 (13.9)
-Saturated (g)	2.9	3.0	2.4
-Unsaturated (g)	0.4	0.6	0.5
-Polyunsaturated	0.2	0.2	0.5
Total carbohydrates (g) (%)	43.2 (73.7)	45.1 (75.1)	41.1 (72.3)
-Free sugars (g)	38.6	38.6	34.7
Dietary fibers (g)	9.2	6.6	8.1
Protein (g) (%)	2.5 (4.3)	2.5 (4.2)	3.7 (6.5)

Abbreviations: FAE: fermented aronia extract, AE: aronia extract.

**Table 2:** Baseline values for participants that have completed the trial. Abbreviations: SD: standard deviation, IQR: interquartile range, W: women, M: men.

Variable (Unit)	Completers (*n* = 23). Value in Mean ± SD or Median (IQR)	Randomized (*n* = 36). Value in Mean ± SD or Median (IQR)
Gender	15 (M) 8 (W)	21 (M) 15 (W)
Age (years)	67.6 ± 5.5	66.9 ± 6.0
Body weight (kg)	82.0 ± 16.2	85.9 (72.6–95.5)
Body mass index (kg/m^2^)	26.7 (23.2–29.8)	28.6 (24.3–32.0)
Waist circumference (cm)	101.5 (94.4–108.2) (M) 115.5 (95.6–124.4) (W)	105.3 (97.1–112.6) (M) 116 (96.8–123.8) (W)
Systolic blood pressure (mmHg)	141 ± 23.3	142 ± 20.9
Diastolic blood pressure (mmHg)	81 ± 13.3	81 ± 11.9
Fasting plasma glucose (mmol/L)	7.7 ± 1.6	7.9 ± 1.8
Hemoglobin A1c (mmol/mol)	50.0 (47.5–54)	50.5 (47.0–55.0)

Abbreviations: SD: standard deviation, IQR: interquartile range, W: women, M: men.

**Table 3:** Overview of participants’ medications.

Medication/Compound	Completers (*n* = 23). Received by (Number Participants (%))	Randomized (*n* = 36). Received by (Number Participants (%))
Metformin	21 (91.3)	33 (91.7)
Insulin	1 (4.3)	3 (8.3)
GLP-1 receptor agonist	4 (17.4)	6 (16.7)
Dipeptidyl peptidase 4 inhibitor	2 (8.7)	2 (5.6)
Sodium–glucose Cotransporter-2 inhibitor	8 (34.8)	9 (25.0)
Sulfonylurea	2 (8.7)	3 (8.3)
Statins	15 (65.2)	25 (69.4)
ACE inhibitor	10 (43.5)	15 (41.7)
Angiotensin II receptor blocker	4 (17.4)	9 (25.0)
Beta-blocker	8 (34.8)	11 (30.6)
Antiplatelet/anticoagulant treatment	6 (26.1)	9 (25.0)
Calcium channel blocker	6 (26.1)	10 (27.8)
Cardiac glycosides	0 (0.0)	1 (2.8)
Diuretics	5 (21.7)	8 (22.2)
Levothyroxine treatment	2 (8.7)	2 (5.6)
Dietary supplement	12 (52.2)	20 (55.6)

**Table 4:** Mean pre- and post-values as well as mean difference (Δ-mean) for anthropometric measurements and blood analyses. Normally distributed data are presented as the mean difference between pre- and post-values ± standard error (Δ-mean). Not normally distributed data are written in italics and presented as ratio (confidence levels), e.g., 1.02 indicates a 2% increase from pre to post, whereas 0.98 indicates a 2% decrease. Means for pre- and post-values are all presented on original scale ± standard error. ^a^ There is a significant increase in BW in the FEA group from pre to post at *p* = 0.04. ^b^ ΔGIP is significantly higher in the AE group than in the placebo group at *p* = 0.03. ^c^ There is a significant increase in GIP in the FEA group from pre to post at *p* = 0.007.

Variable (Unit)		Δ-Mean for FAE		Δ-Mean for AE		Δ-Mean for Placebo	
		Pre	Post	Pre	Post	Pre	Post
Body weight (kg) *n* = 36		0.75 ± 0.30		0.46 ± 0.26		0.60 ± 0.30	
		86.3 ± 4.13 ^a^	87 ± 4.13 ^a^	87.7 ± 4.12	88.2 ± 4.12	87.6 ± 4.12	88.2 ± 4.12
Body mass index (*n* = 36)	*(ratio)*	*1.01 (0.99–1.03)*		*1.01 (0.99–1.03)*		*0.98 (0.96–1.01)*	
	kg/m^2^	28.7	29.0	28.8	29.0	28.9	28.5
Waist circumference (cm) *n* = 36		0.27 ± 0.73		0.54 ± 0.66		−0.16 ± 0.72	
		109.1 ± 3.18	109.4 ± 3.20	109.8 ± 3.18	110.3 ± 3.18	110 ± 3.18	109.9 ± 3.19
HbA1c *n* = 36	*(ratio)*	*1.02 (0.98–1.06)*		*1.02 (0.99–1.06)*		*1.00 (0.97–1.04)*	
	(mmol/mol)	53.8 ± 1.36	54.8 ± 1.42	53.6 ± 1.35	54.8 ± 1.38	54 ± 1.35	54.1 ± 1.39
Fructosamine (µmol/L) *n* = 36		3.90 ± 7.86		12.38 ± 7.09		10.01 ± 7.76	
		294.66 ± 11.08	298.56 ± 11.35	284.84 ± 10.91	297.22 ± 10.91	289.97 ± 10.80	299.98 ± 11.30
Fasting glucose (mmol/L) *n* = 36		0.24 ± 0.26		0.46 ± 0.24		0.24 ± 0.26	
		8.53 ± 0.34	8.77 ± 0.36	8.32 ± 0.34	8.78 ± 0.34	8.38 ± 0.33	8.61 ± 0.35
AUC (mmol/L × 240 min) *n* = 36		−16.76 ± 90.87		55.37 ± 80.70		157.13 ± 88.83	
		3339.12 ± 131.41	3322.36 ± 135.16	3334.96 ± 129.03	3390.33 ± 129.03	3243.17 ± 127.78	3400.30 ± 134.46
iAUC (mmol/L × 240 min *n* = 36	*(ratio)*	*1.00 (0.87–1.14)*		*0.94 (0.83–1.05)*		*1.01 (0.88–1.15)*	
	(mmol/L × 240 min)	1261.33 ± 92.58	1245.19 ± 92.58	1316.59 ± 92.61	1243.24 ± 92.61	1232.13 ± 92.50	1305.22 ± 92.50
Fasting insulin *n* = 23	*(ratio)*	*1.08 (0.91–1.27)*		*0.96 (0.81–1.13)*		*0.89 (0.75–1.05)*	
	(pmol/L)	6.7 ± 1.03	7.22 ± 1.11	6.95 ± 1.07	6.66 ± 1.02	7.48 ± 1.15	6.65 ± 1.02
HOMA-IR *n* = 23	*(ratio)*	*1.09 (0.90–1.32)*		*1.00 (0.83–1.22)*		*0.92 (0.76–1.12)*	
	(response scale)	2.55 ± 0.43	2.78 ± 0.47	2.49 ± 0.42	2.49 ± 0.42	2.73 ± 0.46	2.5 ± 0.43
Matsuda for 120 min AUC (response scale) *n* = 23		−0.27 ± 0.30		0.39 ± 0.30		0.26 ± 0.30	
		4.24 ± 0.66	3.97 ± 0.66	4.02 ± 0.66	4.41 ± 0.66	4.1 ± 0.66	4.35 ± 0.66
Fasting GLP-1 *n* = 23	*(ratio)*	*1.12 (0.86–1.47)*		*1.06 (0.81–1.38)*		*1.01 (0.77–1.32)*	
	(pmol/L)	9.84 ± 2.26	11.05 ± 2.54	9.87 ± 2.27	10.43 ± 2.40	10.19 ± 2.34	10.29 ± 2.36
Fasting GIP *n* = 23	*(ratio)*	*1.59 (1.11–2.29)* ^b^		*0.98 (0.69–1.41)*		*0.94 (0.65–1.34)* ^b^	
	(pmol/L)	5.53 ± 1.01 ^c^	8.81 ± 1.61 ^c^	7.7 ± 1.41	7.57 ± 1.38	6.74 ± 1.23	6.30 ± 1.15
Fasting glucagon (pmol/L) *n* = 23		−0.35 ± 0.46		1.06 ± 0.46		0.54 ± 0.46	
		4.27 ± 0.70	3.92 ± 0.70	2.75 ± 0.70	3.81 ± 0.70	3.71 ± 0.70	4.25 ± 0.70

Abbreviations: FAE: fermented aronia extract, AE: aronia extract, HbA1c: hemoglobin A1c, AUC: area under the curve, iAUC: incremental area under the curve, HOMA-IR: homeostasis model assessment for insulin resistance, GLP-1: glucagon-like-peptide-1, GIP: glucose-dependent insulinotropic polypeptide.

## Data Availability

Data supporting reported results can obtained by contacting the corresponding author.
